# Binding Site Transitions Across Strained Oxygenated and Hydroxylated Pt(111)

**DOI:** 10.1002/open.201800039

**Published:** 2018-05-18

**Authors:** Ian G. Shuttleworth

**Affiliations:** ^1^ School of Science and Technology Nottingham Trent University Nottingham NG11 8NS UK

**Keywords:** hydroxyl, oxygen, platinum, strain engineering, surface chemistry

## Abstract

The effects of strain *σ* on the binding position preference of oxygen atoms and hydroxyl groups adsorbed on Pt(111) have been investigated using density functional theory. A transition between the bridge and FCC binding occurs under compressive strain of the O/Pt(111) surface. A significant reconstruction occurs under compressive strain of the OH/Pt(111) surface, and the surface OH groups preferentially occupy on‐top (bridge) positions at highly compressive (less compressive/tensile) strains. Changes to magnetisation of the O‐ and OH‐populated surfaces are discussed and for O/Pt(111) oxygenation reduces the surface magnetism via a delocalised mechanism. The origins of the surface magnetisation for both O‐ and OH‐bearing systems are discussed in terms of the state‐resolved electronic populations and of the surface charge density.

## 1 Introduction

Pt‐based materials are effective catalysts for the oxygen reduction reaction (ORR).^1^, ^2^ Early experimental reports demonstrated the highly active character of Pt_3_Ni(111)^3^ and across a sequence of related Pt_3_M (M=Ni, Co, Fe, Ti, and V) surfaces.^4^ The ORR mechanism concerns the hydrogenation of O_2_ and mainly occurs along either a four‐electron reduction pathway producing H_2_O or along a two‐electron pathway producing H_2_O_2_. The mechanisms of these pathways, however, are not understood and this lack of understanding is, in part, due to the number of concurrent processes that occur during the reaction. Recent studies of the hydrogenation of O and OH on Pt(111)^5^ have shown that Grotthus^6^ diffusion of H^+^ through aqueous adlayers on the Pt(111) are instrumental in the ORR, but have failed to identify the rate limiting step(s) in the reaction. STM studies^7^ demonstrated the propagation of ‘reaction fronts’ across the Pt(111) surface. However, empirical modelling^7^ of these fronts show significant qualitative disagreements with the experimental data and identify that numerous length‐scale phenomena need to be included in the model to accurately describe the experimental observations. These phenomena include, at larger length scales, OH and H_2_O island formation and, at shorter length scales, the effects of attractive adsorbate‐adsorbate interactions caused by hydrogen bonds between adsorbed molecules.

The mechanism is evidently complex even on a clean, unstrained Pt(111) surface. The experimental observations of reactivity on real (i.e. core–shell nanoparticle) are then further complicated because of the phase segregation in the nanoparticle. This means that the surface of the nanoparticle is typically a pure metal, whereas the bulk of the nanoparticle is an alloy. This causes a lattice mismatch between the surface and the bulk, which will strain the surface. The paradigm of explaining ORR on nanoparticle surface is, therefore, complex. However, to reduce this problem, recent studies have investigated the behaviour of strained bulk alloys, extended phase‐segregated surfaces and strained surface systems. The purpose of the work is to identify the behaviour of O and OH on strained Pt(111) to establish a framework around which the ORR mechanism on strained nanoparticle surfaces can be discussed. The current survey will overview some of the current work in these topics before leading into a more detailed discussion of the O/Pt(111) and OH/Pt(111), which are the subject of the computational sections of this work.

### 1.1 Characterisation of the Pt‐Alloy Bulk and Surface

The studies outlined earlier in this Introduction have focussed on experimental characterisation of the catalysts. A more systematic theoretical approach to understanding why these catalysts work so well is daunting. The principle reasons for these difficulties are the number of degrees of freedom that need to be considered—such as bulk and surface stoichiometry, shape, and topology, and the magnetic character of the system—when investigating a particular group of catalysts.

Recent theoretical studies have started to address these difficulties by surveying the bulk alloy. These investigations have varied the lattice parameter both above and below its equilibrium value and have consequently put the crystal into a state of either tensile or compressive strain, respectively. Changing the lattice parameter in this way will change the interatomic distances within the crystal and the amount of overlap between the electronic orbitals of neighbouring atoms, which consequently will affect the electronic character of the entire system. Investigations into the ordered phases of Ni_*x*_Pt_1−*x*_ (*x=*0.25, 0.5, and 0.75)^8^ and both Pt_*x*_Fe_1−*x*_ and Pt_*x*_Co_1−*x*_ 9 have been recently performed. The investigations focussed on the magnetisation of the unit cell and on the occupancy of the fully state‐resolved atomic orbitals. For each case, the magnetisation was shown to be carried by the Pt and Ni, Fe or Co d states, with all the other states remaining non‐magnetic. The magnetism increased as the strain became increasingly tensile and was accompanied by charge transfer between the magnetic quantum number‐resolved d orbitals. Investigations of strain‐induced changes in the magnetisation of alloys have also been performed on rare‐earth alloys. Studies of the CeNi_5_ system^10^ have shown that the magnetism of the alloy is carried by the Ce f and Ni d states. However, under strain, the spin moment associated with the Ni d states changes far more significantly than those associate with the Ce f states. These observations underlie the importance of the d orbital. The directionality of either the d or f states is evidently insufficient to ensure sensitivity of the spin moment of either to the strain state of the crystal. The radial extent and symmetry of each state must also be factors and may serve as a design criterion for proposed the catalysts.

The technique of ‘strain‐engineering’, or investigating the changes of the character of the system when it is subjected to compressive or tensile strains, mimics the surfaces of core–shell nanoparticles. In the current context, the technique is a simplification to render the systems more accessible to computational study. Including ordered selvedge and bulk alloy layers increases the degrees of structural freedom significantly. Further, Pt_*x*_M_1−*x*_ (M=metal) systems are often experimentally disordered and with a range of stoichiometries.^8^, ^9^ The treatment of alloys with combinations short‐ and long‐range order (i.e. disordered and ordered alloys, respectively) is a well‐known problem; theoretically, statistical treatments^11^ have been applied successfully. Density functional theory (DFT) treatments, however, require a greater level of structural definition and consequently structural approximations are often used. Contemporary DFT studies of Pt_3_M(111) (M=Ag, Au, Co, Cr, Cu, Fe, Ir, Mn, Mo, Ni,Pd, Re, Rh, Ru, Ti, V)^12^ have addressed these problems by simulating five‐layer slabs with a single Pt surface layer and an ordered PtM selvedge region, and the calculated segregation energies were shown to be in agreement with experimental results. Subsequent studies^13^ demonstrated that the oxidisation of the surface disrupts the surface layer though this disruption was characterised more in terms of the segregation energies and layer spacing. This disruption, however, is reflected in the current work particularly in the surface reconstruction seen for OH/Pt(111).

The works discussed in the previous paragraph reduced the nanoparticle surface to a particular facet—notably, the (111)^12^—and then investigated the behaviour of this facet with a pure metal surfaces patterning alloy selvedge and bulk regions. A key design question in this approach is how thick does the pure metal surface covering need to be? This parameter can be controlled both experimentally and in computational treatments. The pure metal surface covering will exhibit strain effects because the selvedge and bulk lattice parameters may be different to the bulk lattice parameter of pure metal, and ligand (i.e. electronic structure) effects due to both the straining of the pure metal and to ‘seeping’ of the alloy wavefunction from regions below the surface. Computational studies of A_3_B (where A=Pt and Pd, and B=Cu, Ag and Au)^14^ and of alloy–core@Pt nanoparticles^15^ have shown that strain effects may persist for surface trilayers though ligand effects are more quickly damped with overlayer thickness and become markedly less significant after single or bilayer surface coverings. Studies of Pt(111) surface layers on Pt_25_Ni_75_(111)^16^ have shown ligand effects are significant for a 2 monolayer (ML) surface Pt thickness, but strain effects dominate for 3–4 ML thicknesses. These observations suggest the range of validity of the approach used in the current work is for Pt overlayers which have thicknesses ≥3 ML.

DFT studies have recently been used to investigate the behaviour of surface H atoms across a sequence of strained Pt^17^ and other pure transition‐metal^18^ slabs. These studies were pertinent not only to the ORR reaction, which has been highlighted in the current work, but also for surface hydrocarbon chemistry^19^ and as a precursor to hydrogen storage.^20^, ^21^ The DFT studies showed that the preferred binding position of H atoms in the H/Pt(111) system can be tuned to be either on‐top or FCC by applying either compressive or tensile strain to the Pt(111) surface.^17^ Similar changes in the preferred H binding site were observed on Pd and Ir for strains of up to 2 % and on Fe, Rh, Ag and Os for strains of up to 5 %.^18^ Consequentially, the reactivity of the H atom may to change under the application strain. This is because the valency between the H atom and the surface will change with binding position, which will redistribute both the charge surrounding the H atom, which is associated with the H‐surface bond, and that which is not. The latter component of charge will be more directly involved in reactions between the H atom and either other adsorbates via the Langmuir–Hinshelwood mechanism or with gas‐phase particles via the Eley–Rideal mechanism.

The sensitivity of H binding position to strain is also a fundamental interest for bulk systems. Studies of the behaviour of the hydrides of alkali and alkaline earth metals under strain^22^ have highlighted the superconducting behaviour of certain alloys under pressure. Evolutionary algorithms have been used to identify a series of novel phases for MH_*n*_ with *n*>1 and M=Li, Na, K, Rb, Cs, and for MH_*n*_ with *n*>2 and M=Mg, Ca, Sr, Ba. In addition, a hexagonal high‐pressure phase of rhodium hydride has recently been predicted by using DFT.^23^ Within the alkaline earth metal hydrides, Mg‐based materials have significant applications in energy storage. The materials have been characterised experimentally and have been seen to exist in rutile,^24^ α‐PbO_2,_ 25 and cubic^26^ phases as well as in two forms of orthorhombic^27^ and in non‐stoichiometric forms.^28^ There clearly is considerable diversity in the structure of hydrides both in bulk form and when patterned on surfaces and the application of this diversity is potentially of great interest.

The phenomenon of H binding site/pressure dependence is, therefore, of current and significant interest, in both applied and fundamental fields. The accompanying fields of the O/Pt(111) and OH/Pt(111) systems under pressure will be addressed in this work. Consequently, Section 1.2. will review these systems.

### 1.2 Oxygenated and Hydroxylated Pt(111)

The interaction of oxygen with Pt(111) has been extensively investigated and a cogent picture of the gas/surface interaction has started to emerge. Early studies^29^ presented the temperature‐dependent behaviour of the interaction, whereby weakly adsorbed molecular oxygen forms on Pt(111) at temperatures below 120 K and the adsorption of molecular oxygen occurs at temperatures in the range 150–500 K and a subsurface oxide forms at temperatures between 1000 and 1200 K. The high‐temperature behaviour was later investigated using surfaces that had been prepared through differing methods of oxidation.^30^ The onset decomposition of the oxygen‐bearing surfaces was observed at 400 K, though no surface oxygen was observed at 1070 K.

The dissociated phase was shown by low‐energy electron diffraction (LEED) to form an ordered p(2×2)‐O layer^29^, ^31^ for coverages up to 0.25 ML. XPS studies^32^ have shown that higher coverage states of oxygen have the same chemical state as those in coverages up to 0.25 ML. It was postulated^33^ that the high coverage state forms through direct dissociation, whereas the lower coverage state forms via a molecularly adsorbed precursor. The similarities in the electronic state of the high and low coverage oxygen states were further evidenced by work function change (Δ*ϕ*) studies,^34^ which showed that Δ*ϕ* varies linearly with oxygen coverage between 0 and 1 ML, which is consistent with the adsorption of a single surface species.

Later studies^35^ demonstrated that a novel high oxygen concentration state could be formed by exposing the Pt(111) surface to thermally cracked oxygen at room temperatures. These finding reflect earlier observations that the maximum oxygen concentration could be increased by exposing the surface to an electron beam^29^, ^31^ or by increasing the surface temperature/oxygen dosing pressure.^32^ The necessity of investigating these dependencies is symptomatic of the need to couple fundamental surface science investigations with those that are performed at more catalytically relevant temperatures and pressure. This can be seen in recent ReactorSTM studies,^36^ which have identified the formation of two high‐coverage surface oxides at high oxygen pressures (up to 5 bar) and surface temperatures of 300–538 K.

Consequently, the current study will focus on oxygen coverages of 0.25 ML. In this phase, the oxygen binding position has been determined experimentally^37^ to be at the FCC site. This binding position has also been predicted by using DFT.^38^, ^39^, ^40^ The current study will also focus on the bonding mechanism and the mechanism of charge relocation that accompany the formation of O<C‐<Pt and OH−Pt bonds. This is in deference to the more common theoretical studies that currently exist in the literature, which have sought to elucidate the mechanisms of phase formation.^38^ The binding mechanism between oxygen and transition‐metal surfaces can proceed through more than one mechanism in deference to the binding mechanism of other molecules such as H,^41^ which binds covalently. This multiplicity has been demonstrated explicitly for O binding to Ni(111) and Ni(100) clusters.^42^ Studies of O/Pt(111)^40^ system have suggested that oxygen binding is accompanied by the exchange of charge between the O p and Pt d orbitals and that there is some suggestion of multiplicity in the bonding mechanism. The current study will investigate these suggested binding mechanisms and also their behaviour as the Pt(111) surface is strained.

Hydroxyl formation on Pt(111) can be achieved by first dosing oxygen onto the clean Pt(111) surface and then reacting the resulting surface with water to form OH/Pt(111).^43^, ^44^, ^45^ Experimentally, however, a pure OH layer cannot be formed,^43^ as hydroxyl is a very good proton acceptor and forms strong OH−H_2_O bonds by H donation from the adsorbed water towards the adsorbed OH group. Theoretical investigations^46^ of low coverages (1/9 ML) of OH have shown that the bridge and on‐top binding sites of the Pt(111) surface are approximately degenerate. The binding energy at these sites is approximately 2.25 eV. Higher coverage theoretical studies^46^ (1/2 to 1 ML) showed that H bonding between adjacent OH groups causes an enhancement of the OH chemisorption energy and a preference for binding at the on‐top site.

Experimental studies^47^ using scanning tunnelling microscopy (STM) and high‐resolution electron energy loss spectroscopy (HREELS) have shown that the on‐top adsorption site is most likely for coverages of 2/3 ML. Consequently, the current work will focus on OH coverages of 0.25 ML, where the formation of a hydrogen‐bond network between adjacent OH groups is anticipated to be minimal. Analysis of the OH bending mode in the HREELS investigations^47^ showed that the OH molecule is tilted from the surface normal. Tilting has been discussed theoretically with early DFT studies,^48^, ^49^ indicating that OH tended to bind in an upright position in hollow sites whereas tilting is seen to lower the binding energy when the OH is bound to the on‐top or bridge sites.^50^ In the current work the degree of tilt of the OH group and the orientation of the OH bond with respect to the Pt(111) surface will be allowed to fully relax.

In the remainder of this work, the computational details are outlined and then investigations of the strained extended surface O/Pt(111) and OH/Pt(111) systems are presented in turn and discussed. The work will then conclude with a presentation of the key findings of these computational investigations.

## Computational Details

The DFT simulations presented in this work were performed using the plane‐wave Quantum Espresso package.^51^ The kinetic energy cut‐offs for wave‐functions and for charge density and potential were set to 75 and 300 Ry, respectively. Brillouin zone integration was performed on a (6×6×1) grid using a first‐order Methfessel–Paxton^52^ smearing of 0.02 Ry. The PBE exchange correlation functional within the generalised gradient approximation (GGA) was used throughout this work and the core electrons were described with norm‐conserving pseudopotentials.^53^ Using this approach, the equilibrium bulk lattice constant of Pt was determined to be *L*_0_=3.980 Å, which is comparable to the experimental value of the lattice constant of 3.92 Å. All results presented in the current work were obtained by using fully spin‐polarised simulations. The van der Waals correction has not been included in the current study as the focus of the work is on the O−Pt bond at low O and OH coverages. The van der Waals interaction might have a greater significance for higher coverages, particularly in regimes where the H atoms are more closely bound to other surface species. The assumption of low surface coverage is to simplify the system and to ensure that the focus of the current study is on the O−Pt bond and its behaviour under strain. The current study consequently excludes the complicating effects of lateral (adsorbate‐adsorbate) interactions. Effects such as the presence of water molecules in the vacuum region are similarly excluded for the same reason; inclusion of greater particle densities would be more appropriate to molecular dynamics for which the focus is not on the behaviour of the adsorbate‐substrate bond, or the binding position of the adsorbate.

The surface was modelled using slabs containing seven layers of Pt atoms. Subsequent slabs were separated by a vacuum with a width of approximately ten lattice constants. During relaxation, the central layer of Pt atoms were fully constrained. The remaining atoms were allowed to relax freely. Compressive and tensile strains were applied by changing the lattice constant *L* by using Equation (1):

(1) $$L=\left(1+\sigma \right)L_{0}$$

where *σ* is the strain and $\sigma \in \left[-0.05,+0.05\right]$ . By using this convention, the applied strain was termed tensile (compressive) for *σ*>0 (*σ*<0). Equation (1) consequently defines the distance between Pt atoms in the central layer of each slab at the beginning of each simulation and this distance was not subsequently changed during the simulation.

**Figure 1:**
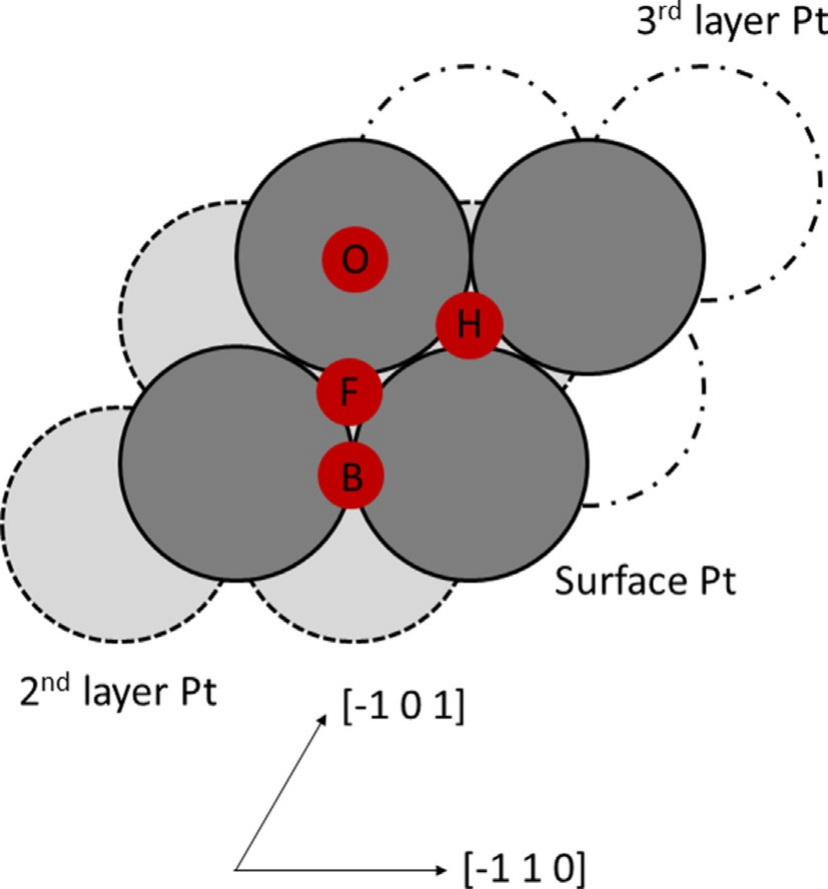
Oxygen and hydroxyl binding positions across the Pt(111) surface. The small red circles denote the binding positions of the oxygen atoms and hydroxyl groups and the labels ‘H’, ‘F’, ‘O'and ‘2’ denote the HCP, FCC, on‐top and two‐fold bridge sites, respectively. The large dark grey, light grey and white circles denote Pt atoms in the surface, second and third Pt layers, respectively.

Figure 1 shows the binding positions within the unit cell with the O atoms or OH groups bound in equivalent positions on either side of the slab. (2×2) Surface supercells were used for each simulation, and each of these supercells contained only a single O atom or OH group. This low concentration (0.25 ML) reduces the effect of lateral interactions between the adsorbates, and at this concentration no evidence was found of H transfer between adjacent OH groups.

The O and OH binding energies *E*_B_ were defined by Equation (2):

(2) $$E_{B}=\frac{1}{2}\left(E_{Ads/Pt}-2E_{Ads}-E_{Pt}\right)$$

where $E_{Ads/Pt}$ is the total energy of the O or OH bearing (2×2)‐Pt(111) slab, $E_{Ads\;}$ is the total energy of an isolated O atom or OH group, and $E_{Pt}$ is the total energy of a clean, fully relaxed (2×2)‐Pt(111) slab. The factors of 2 and $1/\;2$ account for the binding of O atoms/OH groups on either side of the slab.

To characterise electronic changes to the surfaces under strain, the projected density of states (PDOS) curves for the p components of the surface O atoms and the d components of their nearest Pt atoms were analysed by calculating the magnetic quantum number resolved occupancy *N*_*l,m*_ using Equation (3):

(3) $$N_{l,m}=\underset{-\infty }{\int^{E_{F}}}g\left(l,m,m_{s},\sigma ,E\right)dE$$

where $g\left(l,m,m_{s},\sigma ,E\right)$ is the spin‐resolved PDOS, *l*,*m* and *ms* are the angular, magnetic and spin quantum numbers, respectively, and *E* is energy. To reduce the analysis the angular quantum number resolved occupancy was calculated by using Equation (4):

(4) $$N_{l}=\sum_{m}N_{l,m}\left(l,m,m_{s},\sigma \right)$$

The analysis procedure outlined in Equations (3) and (4) will characterise the electronic state of the system in energy space. To obtain a real‐space characterisation the difference electron density *ρ*_diff_ was calculated by using Equation (5):

(5) $$\rho_{diff}\left(\sigma ,\;x_{\sigma }\right)=\rho \left(\sigma \neq 0,\;x_{\sigma }\right)-\rho \left(\sigma =0,x_{\sigma }\right)$$

where *ρ* is the electron density of the strained (*σ*≠0) or unstrained (*σ*=0) system. $x_{\sigma }$ is the normalised position coordinate, and is defined by Equation (6):

(6) $$x_{\sigma }=\frac{x}{\left(1+\sigma \right)}$$

x is the absolute coordinate used during the calculation of *ρ*.

The work function *ϕ* of the oxygen‐ or hydroxyl‐covered slab was calculated by using Equation (7):

(7) $$\varphi =V\left(\infty \right)-E_{F}$$

*E*_F_ and *V(∞)* are the Fermi energy and the potential at a height of five lattice constants above the slab, respectively. The work function of the clean Pt(111) slab was defined in a similar way but is denoted *ϕ*_111_.

## 2 Results and Discussion

### 2.1 O/Pt(111)

**Figure 2:**
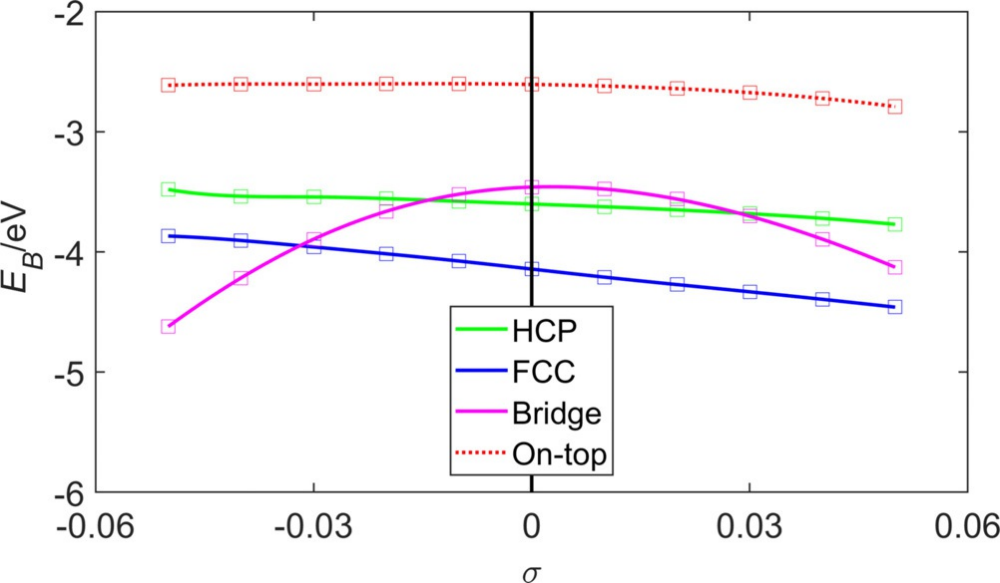
Oxygen binding energy *E*_B_ as a function of strain *σ* for the (2×2)‐O/Pt(111) system. The legend indicates the binding position of the oxygen atom, and these binding positions are shown in Figure 1. The solid/dotted lines are a guide for the eye.

Figure 2 shows the behaviour of the oxygen binding energy *E*_B_ as a function of strain *σ* for oxygen atoms bound in the HCP, FCC, bridge and on‐top positions. The curves show that at equilibrium (*σ*=0) the preferred binding position of the oxygen atom is at the FCC position. This is in agreement with previous experimental^38^ and theoretical DFT^38^, ^39^, ^40^ investigations. The curves also show that, as the strain becomes increasingly compressive (*σ*<0), the preferred binding position changes from the FCC to the bridge position and that this transition occurs at *σ*=−0.03. The O−Pt bond length for FCC‐ and bridge‐bound O was 2.07 and 2.02 Å, respectively, for the unstrained (*σ*=0) surface, and varied by <0.01 Å from these values as *σ* was varied across the interval [−0.05,+0.05].

**Figure 3:**
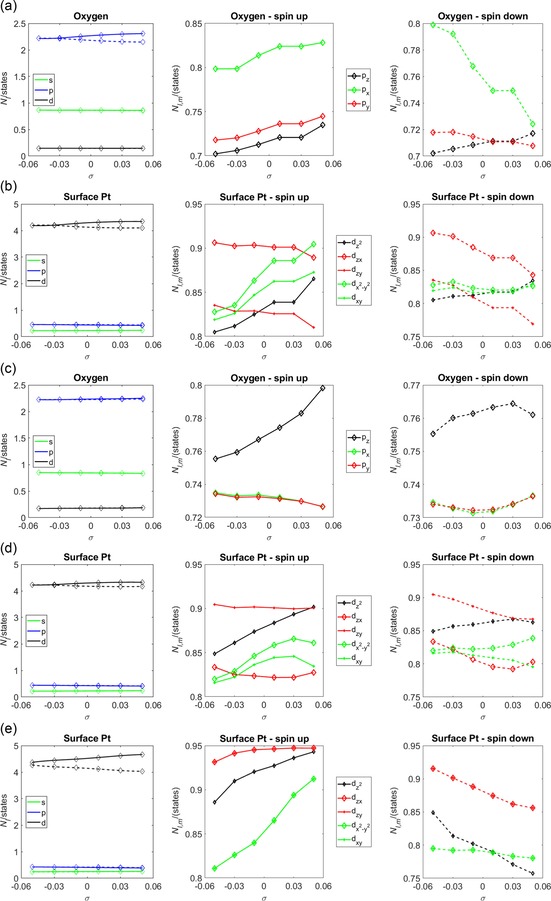
The angular and magnetic quantum number resolved O and their nearest‐neighbour surface Pt state populations, *N_l_* and *N*_*l*,*m*_, respectively, shown as a function of strain *σ* for the (2×2)‐O/Pt(111) system. The panels show these populations for the oxygen and their nearest neighbour surface Pt atoms for systems, where the oxygen atoms are bound in a, b) the two‐fold bridge and c, d) the FCC sites. e) The state populations of the surface Pt atoms for the clean Pt(111) system. The spin (down) components are shown as solid (dashed) lines.

The changes in the surface geometry discussed in the previous paragraph are accompanied by changes in the surface electronic structure. Figure 3 shows the angular and magnetic quantum number resolved oxygen and surface Pt state populations, *N_l_* and *N*_*l,m*_, respectively, as a function of strain *σ* for the (2×2)‐O/Pt(111) system. The *N_l_* shown in Figures 3 a–e show that the strain‐dependent magnetisation of the surface layers develops in the O p and Pt d states. Consequently, discussion of the *N*_*l,m*_ will focus on the states. The occupation of the spin and down components are linked to the magnetic moment on each atom from the relationship that the magnetic moment equals difference between the total spin up and the total spin down charge.

Bridge‐bound oxygen is energetically preferred for *σ*≤−0.03. The *N_l_* in Figure 3 a show that in the *σ*>−0.03 interval the O p states become spin split and within the *σ*≤−0.03 interval the O p states are non‐magnetic. This behaviour is reflected in the behaviour of the *N_l_* of the Pt d states shown in Figure 3 b, which show a transition between zero and non‐zero spin splitting at *σ=*−0.03.

FCC‐bound oxygen is energetically preferred for *σ*>0.03. However, the *N_l_* in Figure 3 c show that across the entire range of *σ* the O p states undergo considerably less spin splitting than their bridge‐bound counterparts. Equivalently, Figure 3 d shows that the *N_l_* of the Pt d states spin split above *σ=*−0.03, but less than their counterparts in the bridge bound case shown in Figure 3 b.

These observations indicate that the local magnetic moment of the surface oxygen atoms and their nearest‐neighbour surface Pt atoms are dependent on the amount of strain *σ* applied to the surface. To elucidate the origin of this change in the local magnetic moment, Figure 3 e shows the *N_l_* of the clean surface Pt atoms. In this case, there is very clear spin splitting across the entire range of *σ*. The amount of spin splitting is also very clearly greater than the amount of Pt d spin splitting seen at equal *σ* for each of the oxygenated surfaces. The development of a local Pt d state spin moment under *σ* is, therefore, a consequence of the application of strain to either the clean or the oxygenated surfaces. The Pt d magnetisation is reduced by the presence of surface oxygen. The O p state magnetisation has been shown to minimise under *σ* as the energetically preferred binding positions tend to carry low oxygen magnetic moments.

To quantify the amount of intra‐orbital charge transfer that accompany these changes in *N_l_*, Figure 3 shows the magnetic quantum number resolved O and their nearest‐neighbour surface Pt state populations *N*_*l,m*_ for O atoms bound in Figure 3 a and 3 b bridge, and Figure 3 c and 3 d on‐top sites. For comparison, the *N*_*l,m*_ for surface Pt atoms on clean Pt(111) is shown in Figure 3 e. Figure 3 a shows that the magnetic moment localised on the O atoms when bound in the bridge site increases as the population of each of the spin up component of the O p_z_, p_x_ and p_y_ states increases and, most significantly, the spin down component of the O p_x_ state decreases. For O bound in the FCC site, the magnetic moment localised on the O atom is relatively small when compared with the bridge‐bound O magnetic moment shown in Figure 3 a and the Pt magnetic moments shown in Figure 3 b–3 e. However, a moment does develop as the strain *σ* becomes increasingly tensile above *σ*=−0.03 and is predominantly attributed to increases (decreases) in the spin up components O p_z_ (p_x_ and p_y_) state(s), and increases in the spin down component of the O p_z_, p_x_ and p_y_ states.

Comparison of *N_l_* in Figures 3 a–3 e shows that, for the lowest energy structure at each *σ*, the surface magnetisation is predominantly attributed to the Pt atoms. Furthermore, comparison of the Pt *N_l_* in Figures 3 b, 3 d and 3 e shows that O adsorption tends to reduce the Pt moments. Figure 3 e shows that for the clean Pt(111) surface, the Pt d_xy_ and $d_{x^{2}{-y}^{2}}$ states and the Pt d_zy_ and d_zx_ states are degenerate. Figures 3 b and 3 d show that this degeneracy is lifted by the presence of surface oxygen. The degree of splitting (i.e. the energetic difference between the Pt d_xy_ and $d_{x^{2}{-y}^{2}}$ states, or the energetic difference between the Pt d_xy_ and $d_{x^{2}{-y}^{2}}$ states) is approximately monotonic between the bridge‐bound O (Figure 3 b) and FCC‐bound O (Figure 3 d). This is highly suggestive that the mechanism for splitting is delocalised, as a localised or directional mechanism would be expected to change as the binding position changed from two‐ to three‐fold. Figure 3 d also shows quantitatively that, within the energetically preferred region where the magnetic moment is significant (i.e. when the O is FCC bound and *σ*≥−0.03), the largest changes in spin up populations are to the Pt $d_{z^{2}}$ state. Lesser, though comparable, changes affect the spin up Pt d_xy_ and $d_{x^{2}{-y}^{2}}$ states, whereas the largest changes in the spin down population are to the Pt d_zy_ and d_zx_ states. The significance of these latter qualitative differences is not yet fully realised, but it may be conjected that spin‐polarised injections to the surfaces may be an effective mechanism of inducing either mechanical or electronic strain.

**Figure 4:**
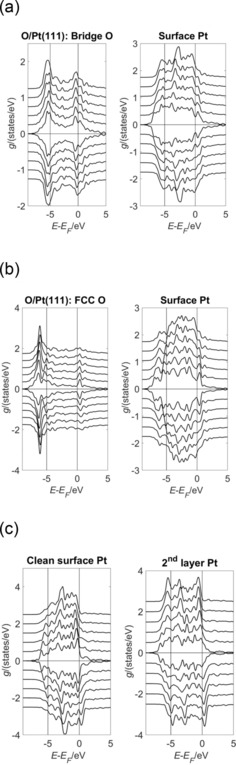
Projected density of states (PDOS) *g* of the O p and the nearest‐neighbour surface Pt d states shown as a function of both energy (*E*−*E*_F_) and strain *σ* for the (2×2)‐O/Pt(111) system. Each graph shows the spin‐up and spin‐down components in the *g*>0 and *g*<0 portions of that graph, respectively. Subsequent curves are offset vertically for clarity and were obtained by using values of *σ*=0.95, 0.97, 0.99, 1.01, 1.03 and 1.05. The curves corresponding to *σ*=0.95 (1.05) curves lie closest to (farthest from) the line *g=*0. The oxygen atoms were bound in a) the two‐fold bridge and b) the FCC positions defined in Figure 1. c) The PDOS of the surface and second‐layer Pt d states for clean Pt(111).

The discussion so far has focused on the *σ*‐dependent changes to the surface structure of the O/Pt(111) system and the accompanying changes in the surface magnetisation. Figure 4 a–c shows the projected density of states (PDOS) *g* for the O p states and the nearest‐neighbour surface Pt d states for the bridge‐ and FCC‐bound O/Pt(111) and clean Pt(111) surfaces, respectively. ‘Nearest‐neighbour’ denotes the Pt atom that is nearest to the surface O atom. The O s states are significantly populated, but are at energies *E*−*E*_F_ ≈ −20 eV, so do not contribute significantly to the O−Pt d‐state bond.

For the bridge‐bound O/Pt(111) system, Figure 4 a shows the bonding (antibonding) O p states in a band spanning *E*−*E*_F_ ≈ −6 eV to −4.5 eV (*E*−*E*_F_ ≈< M‐>1 eV to +2 eV). The occupation of the spin up O antibonding state increases with *σ*. This can be inferred qualitatively from Figure 4 a, but is shown quantitatively in the O spin up *N*_*l,m*_ panel of Figure 3 a. The occupation of the spin down O p antibonding state decreases with *σ* and this change can be seen qualitatively and quantitatively in the same way. There is a net increase in the antibonding population, and this increase weakens the O−Pt bond and contributes to the decreases in the magnitude of the oxygen binding energy *E*_B_ for the bridge‐bound curve shown in Figure 2. These decreases contribute to the shift in preferred binding position from the bridge to the FCC site as *σ* increases.

The bonding and antibonding O p states occupy similar energy bands for the FCC‐bound O/Pt(111) system. This is shown in Figure 4 b. Significantly, the antibonding state for the FCC‐bound O/Pt(111) system does not span the Fermi level (*E*−*E_F_*=0 eV) in the way seen for the bridge‐bound O/Pt(111) system. Consequently, its occupation is not sensitive to *σ*‐dependent changes in the Fermi level in the way that the state was sensitive for the bridge bound case. The population of both the spin up and down O p bonding states increase as *σ* increases. This can be seen qualitatively by the height of these states in Figure 4 b and quantitatively by the *N*_*l,m*_ in Figure 3 c. This strengthens the O−Pt bond and causes the increases in the magnitude of the oxygen binding energy *E*_B_ for the FCC curve shown in Figure 2.

In both the bridge‐ and FCC‐bound cases, the nearest‐neighbour surface Pt atom develops d state density in the energy intervals spanned by the bonding and antibonding O p states. This can be seen by comparing either of the Pt panels of Figure 4 a and 4 b with the Pt panel of Figure 4 c, and is indicative of a covalent interaction between the O and Pt states across these energies. Figure 4 c shows that the clean surface Pt d states become narrower as the strain *σ* becomes increasing tensile. This narrowing is not seen for the second layer Pt atoms, which are also shown in Figure 4 c and shows that the surface layer becomes less metallic as *σ* increases. The formation of bonding O−Pt interactions across *E*−*E*_F_ ≤ −4.5 eV reverses this trend around the nearest‐neighbour surface Pt site for the range of tensile *σ* investigated in the current work. However, at higher values of tensile *σ*, the formation of bonding O−Pt interactions may become problematic because of this tendency of the clean surface Pt d states to narrow. Bulk‐bound oxygen may be less affected.

**Figure 5:**
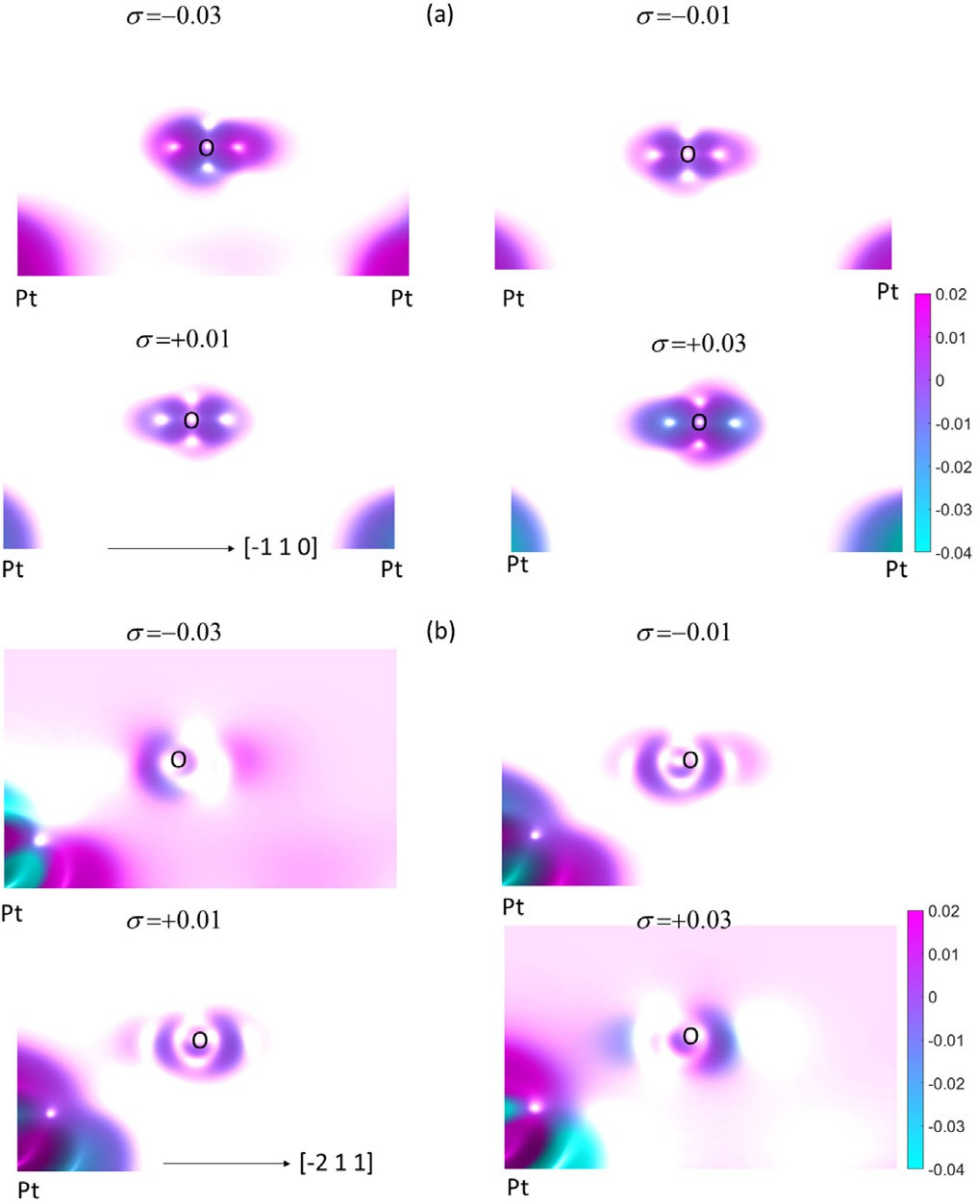
The difference electron density *ρ*_diff_ for the (2×2)‐O/Pt(111) system for strain *σ*=−0.03, −0.01, +0.01 and +0.03. The oxygen atoms are bound in a) the two‐fold bridge and b) the FCC sites. The crystalline direction shown in the bottom left of each panel is the same for each plot within that panel. The legend shows the difference electron density in units of electrons/Ry^3^.

Figure 5 shows the difference electron density *ρ*_diff_ for the (2×2)‐O/Pt(111) system with the O atoms bound in the two‐fold bridge and the FCC sites. For Figure 5 a, the O atoms are bound in the two‐fold bridge site and *ρ*_diff_ shows that charge accumulates in lobes whose axes are perpendicular to the Pt surface for tensile strains of *σ*=+0.01 and +0.03, and are depleted from the surface‐parallel nodes. These changes are reflected in changes to the *N*_*l,m*_ for the bridge‐bound O/Pt(111) system shown in Figure 3 a, where an increase (decrease) in the total population of the O p_z_ (p_x_) states is observed as *σ* becomes increasingly tensile. A more nominal change is noticed in the population of the O p_y_ state.

For Figure 5 b, the O atoms are bound in the FCC site and *ρ*_diff_ shows that charge transfer occurs predominantly between lobes whose axes are parallel to the Pt surface for all strains. Charge accumulation is observed for *σ*=−0.03 in node closest to the O‐nearest‐neighbour surface Pt bond. This node, however, becomes depleted as *σ* increases and charge accumulation occurs on the opposite surface parallel node. These charge transfers are accompanied by increases in the *N*_*l,m*_ for the O p_z_ states shown in Figure 3 c. Consequently, it has been seen that the effect of increasing *σ* on *ρ*_diff_ is to increase the charge density in lobes centred on the O atom and whose axes are perpendicular (parallel) to the Pt(111) surface for the bridge‐ and FCC‐bound systems, respectively. Occupation of the O p_z_ state increases as the strain becomes increasingly tensile and charge accumulation favours directions that are aligned with the O‐nearest‐neighbour Pt direction for increasingly compressive strains.

**Figure 6:**
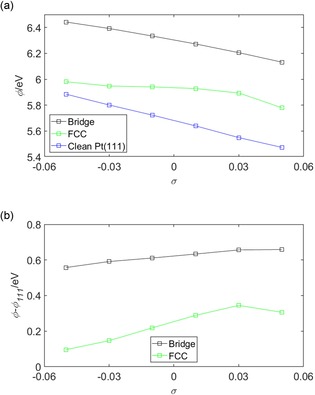
a) The work function *ϕ* of the (2×2)‐O/Pt(111) system and b) the work function difference *ϕ*−*ϕ*_111_, where *ϕ*_111_ is the work of the clean Pt(111) surface, shown as a function of *σ*. The legends in (a) and (b) show the binding position of the oxygen atom across the oxygenated surfaces at either the ‘Bridge’ or ‘FCC’ sites (defined in Figure 1) or indicates, in (a), the curve corresponding to the clean Pt(111) surface.

Figure 6 shows the work function *ϕ* and the work‐function difference *ϕ‐ϕ*_111_, where *ϕ*_111_ is the work of the clean Pt(111) surface, of the (2×2)‐O/Pt(111) system under strain. Changes in *ϕ* owing to *σ* will have two components. The first component is attributed to Smoluchowski smoothing.^54^ This smoothing reduces the amplitude of the Pt surface wavefunction as the surface lattice constant increases, that is, as *σ* becomes more tensile. This effect can be seen clearly in Figure 6 a, as the clean Pt(111) curve shows a decrease in *ϕ* as *σ* increases. Mechanistically, this reduction occurs because, as the surface wavefunction becomes smoother, the accompanying surface dipole and, equivalently, the potential barrier to removal of an electron from the bulk of the crystal to the vacuum, reduces. The second contribution to the changes in the *ϕ* is attributed to the charge redistribution shown in terms of *ρ*_diff_ in Figure 5. To evaluate the relative importance of these two contributions the work‐function difference *ϕ*−*ϕ*_111_ was calculated and is shown in Figure 6 b. The changes caused by the difference *ϕ*−*ϕ*_111_ are predominantly attributed to changes in *ρ*_diff_ and change by ≈+0.1 and +0.2 eV for the bridge‐ and FCC‐bound systems, respectively, as *σ* changes from −0.05 to +0.05. Across the same strain interval, *ϕ* changes by approximately −0.25 and −0.2 eV for the bridge‐ and FCC‐bound systems, respectively, and clearly shows that the effects are comparable.

### 2.2 OH/Pt(111)

**Figure 7:**
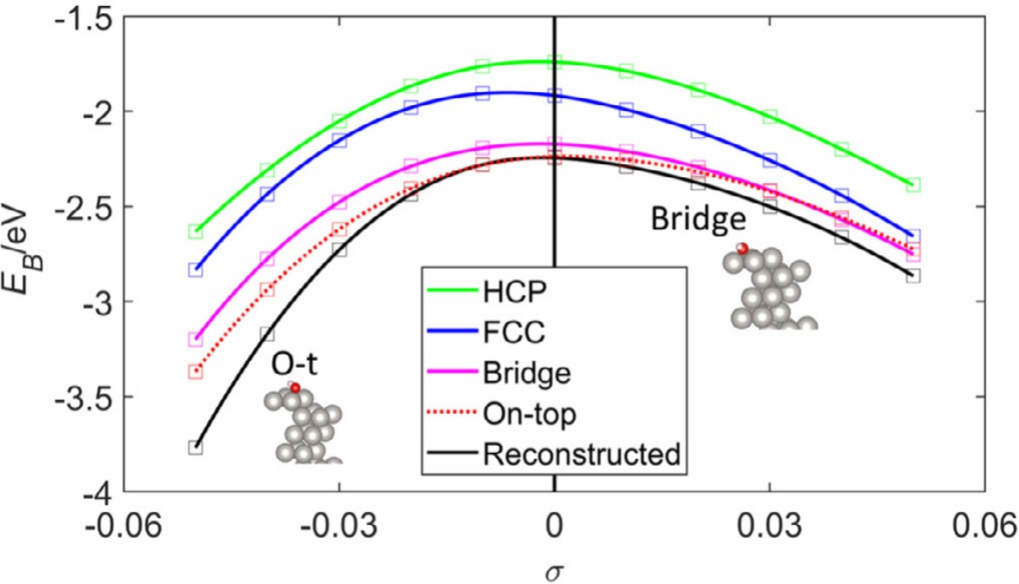
Hydroxyl (OH) binding energy *E*_B_ as a function of strain *σ* for the (2×2)‐OH/Pt(111) system. Binding energies *E*_B_ for both the reconstructed and unreconstructed Pt surfaces are shown. The insets show the OH groups bound to the reconstructed Pt surface in either the on‐top (O‐t) or the two‐fold bridge (Bridge) positions, which occur for *σ*< −0.01 and *σ* ≥ −0.01, respectively (see text). The legend denotes the binding energies *E*_B_ for OH bound to the unreconstructed Pt surface in the ‘HCP’, ‘FCC’, ‘Bridge’ and ‘On‐top’ positions. The solid/dotted lines are a guide for the eye.

Figure 7 shows the hydroxyl (OH) binding energy *E*_B_ as a function of strain *σ* for the (2×2)‐OH/Pt(111) system. The curves in Figure 7 for binding of OH in the unreconstructed HCP, FCC, bridge and on‐top positions were obtained by initially relaxing the OH group above the binding position with constraints applied to the Pt and O atoms that prevented motion parallel to the (111) plane. Once a local minimum had been approximately found by using these constrained minimisations, the local minimum was more accurately determined by removing the (111)‐directed constraints to the Pt and O atoms and restarting the minimisation process.

**Figure 8:**
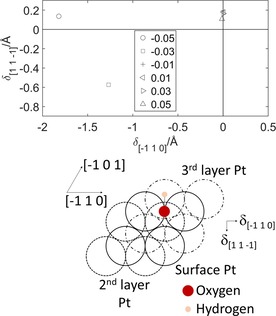
Reconstruction of the (2×2)‐OH/Pt(111) system as a function of strain *σ*. The schematic diagram shows the surface, second‐ and third‐layer Pt atoms of the unreconstructed surface and the displacement vectors *δ*_[−1 1 0]_ and *δ*_[1 1 −1]_, which are directed along the [−1 1 0] and [1 1 −1] directions, respectively. The graph shows the values of the displacement vectors for strains *σ* between ±0.05 and the legend indicates the strain *σ* for each datum.

However, measurements during the determination of the unreconstructed curves outlined in the previous paragraph showed that a global minimum could be obtained by allowing the Pt atoms to reconstruct parallel to the (111) plane. To investigate this reconstruction, the OH group and surface Pt atoms were initially displaced along the orthogonal [−1 1 0] and [1 1 −1] directions before relaxation, and then were not constrained during the subsequent relaxation. These crystalline directions and the displacement vectors *δ*_[−1 1 0]_ and *δ*_[1 1 −1]_ are shown in Figure 8. A full sampling of the possible reconstructions was determined by increasing the initial displacements *δ*_[−1 1 0]_ and *δ*_[1 1 −1]_ incrementally four times across the unit cell, resulting in (4+1) × (4+1)=25 relaxations for OH bound in each of the high symmetry sites.

The resulting reconstruction is shown in Figure 8. The Pt atoms reconstruct predominantly along the [−1 1 0] direction for large compressive strains (*σ*=−0.05) and along both the [−1 1 0] and [−1 1 0] directions for smaller compressive strains. The magnitude of the reconstruction along the [−1 1 0] for large strains is comparable to the Pt–Pt distance in that direction (2.814 Å). At larger compressive strains (*σ*<−0.05), a significant out‐of‐plane motion of the surface Pt atoms developed. Quantification of this reconstruction was not straightforward as, because of the magnitude of the out‐of‐plane motion of the Pt atoms, the reconstruction began to extend across surface distances greater than those described by a (2×2) unit cell. The O−Pt interaction is consequently more significant the Pt–Pt interaction between the surface and second layers for compressive strains of up to *σ*=−0.05, and becomes increasingly more significant that the interaction between adjacent surface Pt atoms for more compressive strains. On the reconstructed surface, the O−Pt bond lengths were 2.07–2.05 Å for $\sigma \in \left[-0.05,-0.03\right]$ (on‐top binding) and 2.21–2.19 Å for σ∈(-0.03,+0.05] (bridge binding). On the non‐reconstructed surfaces, the O−Pt bond lengths were 2.02 and 2.15 Å for binding in the on‐top and bridge positions, respectively. These O−Pt bond lengths varied by <0.01 Å from these values as *σ* was varied across the interval [−0.05,+0.05].

**Figure 9:**
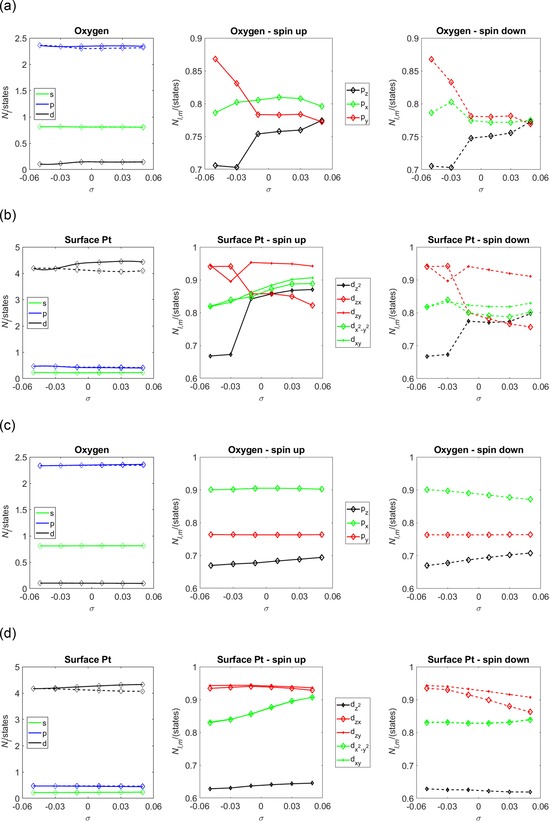
The angular and magnetic quantum number resolved O and their nearest‐neighbour surface Pt state populations, *N_l_* and *N*_*l*,*m*_, respectively, shown as a function of strain *σ* for the (2×2)‐OH/Pt(111) system. The panels show these populations for the oxygen and their nearest neighbour surface Pt atoms for systems a b) where the OH groups are bound across the reconstructed OH/Pt(111) system and c, d) where the OH groups are bound in the on‐top sites of the unreconstructed Pt(111) surface. The spin (down) components are shown as solid (dashed) lines.

Figure 9 shows the angular and magnetic quantum number resolved oxygen and surface Pt state populations, *N_l_* and *N*_*l,m*_, respectively, as a function of strain *σ* for the (2×2)‐OH/Pt(111) system. Figures 9 a and 9 b show these populations for the reconstructed OH/Pt(111) system and Figures 9 c and 9 d show the populations for the on‐top bound OH/Pt(111) system, which are included to highlight differences between populations of the on‐top position on both reconstructed and non‐reconstructed Pt surfaces. In the reconstructed system, the OH group is bound at the on‐top position for strains *σ*<0.01 and is bound at the bridge position for strains *σ*≥0.01. Consequently, there should be some comparison between the orbital populations of the reconstructed and non‐reconstructed surfaces, which may distinguish between general trends and those that are more closely related the preferred site occupancy.

Figure 9 shows a splitting in the *N_l_* of the O p and Pt d states, which is similar to that seen for the O/Pt(111) system and shown in Figure 3. This shows that qualitatively the presence of H does not prevent the development of magnetism. This is a common phenomenon and is attributed to the delocalised H s states. However, the Pauli effect between the O and their nearest‐neighbour Pt atoms is evidently stronger than this H damping effect for these OH/Pt(111) systems. Figures 9 a and 9 b show a clear transition in *N*_*l,m*_, as the surface undergoes the structural transition between on‐top bound OH (*σ*<0.01) and bridge bound OH (*σ*≥0.01). Figure 9 a shows that this transition is accompanied by a significant decrease (increase) in the population of the oxygen p_y_ (p_z_) state for both spin components as strain *σ* becomes increasingly tensile. Figure 9 c shows a similar trend in the O p_z_ state for on‐top bound OH. However, in this latter case the changes in the O p_z_, p_x_ and p_y_ states are comparatively smaller.

Consequently, the O p_z_ state is generally electrophilic as *σ* becomes increasingly tensile, and changes in the valence of the OH group as it moves from on‐top to the bridge site increase this tendency. Smoluchowski^54^ smoothing arguments would suggest that as *σ* and the surface lattice constant increases the Pt component of the surface wavefunction will smooth and less charge from this wavefunction would encroach on the O atom. This suggests that, electrostatically, the population of the O p_z_ state is limited by the presence of Pt charge from the surface wavefunction. Sterically, the O atom increases its valence with the surface Pt atoms in the bridge position compared to its valence in the on‐top position. This increase in valence is accompanied by charge flow from the O p_y_ state into the O p_z_ state. By comparing Figure 9 a and 9 c, it is clear that the charge transfers into the O p_z_ state from the O p_y_ state, which is enabled by the steric changes on the surface that occur as the OH migrates to between the bridge and the on‐top position is central to the strain‐dependant character of the OH/Pt(111) system.

Figure 9 b shows a clear transition in the *N*_*l,m*_ for both spin components of the nearest‐neighbour Pt $d_{z^{2}}\;$ and d_zx_ states as the surface undergoes the structural transition between on‐top bound OH (*σ*<0.01) and bridge‐bound OH (*σ*≥0.01). *N*_*l,m*_ for the clean Pt surface have been presented in Figure 3 e and show degeneracy between the Pt d_xy_ and $d_{x^{2}-y^{2}}\;$ states as well as the Pt d_zx_ and d_zy_ states for all *σ*. These degeneracies are lifted on the reconstructed OH/Pt(111) surface (Figure 9 b), though only the Pt d_zx_ and d_zy_ degeneracy is lifted for the unreconstructed OH/Pt(111) surface, as shown in Figure 9 d.

To distinguish between effects that are caused by the Pt reconstruction and those that are from the OH group, the *σ*<0.01(compressively strained) portions of Figures 9 b and 9 d are compared. The reason for this comparison is becuase the OH group is binding in the on‐top position for both the reconstructed and the non‐reconstructed surfaces in this strain interval. Figure 9 b shows that the reconstruction lifts the degeneracy between the Pt d_xy_ and $d_{x^{2}-y^{2}}\;$ states as well as the Pt d_zx_ and d_zy_ states when compared to Figure 9 d, which shows a lesser lifting between the Pt d_zx_ and d_zy_ states and no lifting of the degeneracy between the Pt d_xy_ and $d_{x^{2}-y^{2}}$ states.

Generally, the changes in registry between the surface and second‐layer Pt atoms accompanying the reconstruction may be expected to change the symmetry of the surface wavefunction and effect the degeneracy changes. The magnitude of the energy differences between the Pt d_zx_ and d_zy_ states and the Pt d_xy_ and $d_{x^{2}-y^{2}}\;$ states would not be directly estimated by using a purely symmetric argument, but are now quantified in Figure 9 b. However, the amount of degeneracy lifting in the less compressive (*σ*≥0.01) region is affected by the binding position of the OH group, particularly for the Pt d_zx_ and d_zy_ states whose separation in the *σ*≥0.01 region is significantly more than for *σ*<0.01.

Overall, the effect of the change in binding position of the OH group from the on‐top to the bridge site is to increase the occupancy of the Pt $d_{z^{2}}\;$ and reduce the population of the Pt d_zx_ states for both spin polarisations. The change in the binding position also decreases the level of the degeneracy between the Pt d_zx_ and d_zy_ states and, to a lesser extent, the the Pt d_xy_ and $d_{x^{2}-y^{2}}\;$ states. Changes in the registry between the surface and second‐layer Pt atoms during the reconstruction also contributes to this loss of degeneracy, but to a lesser extent.

**Figure 10:**
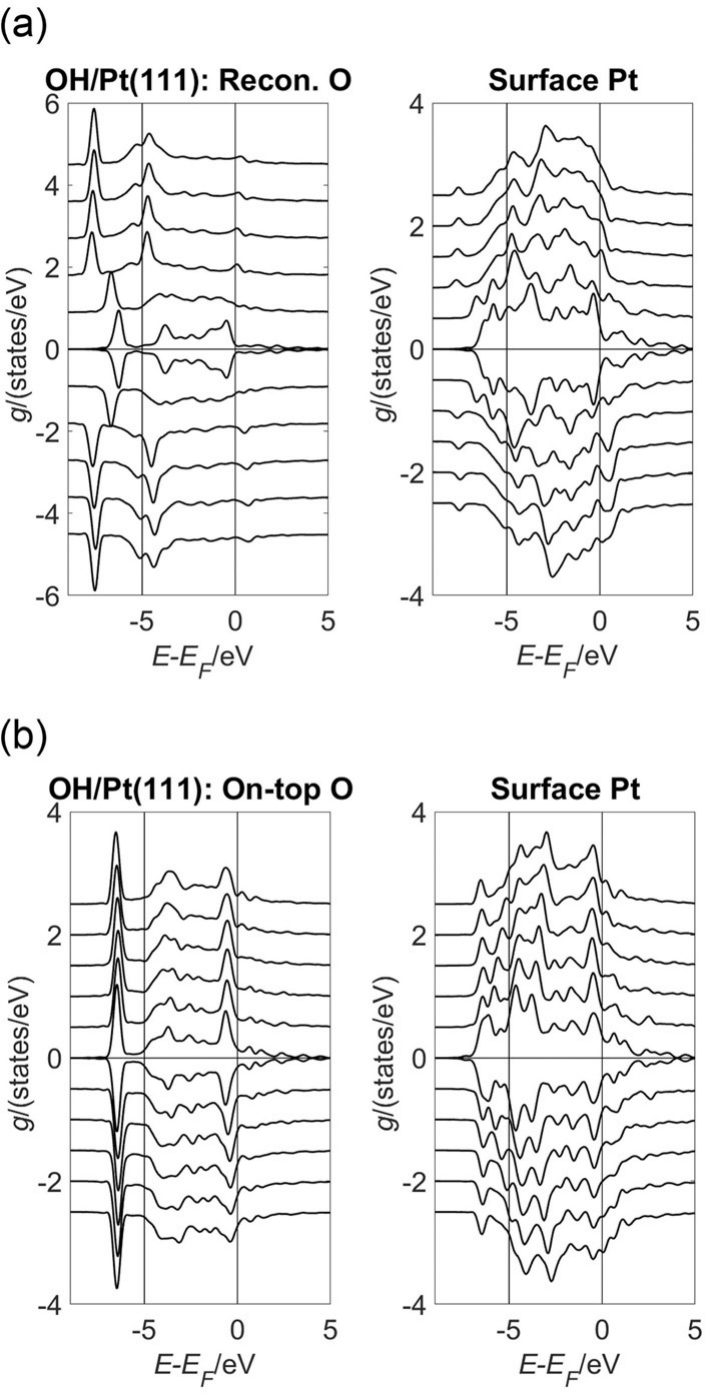
Projected density of states (PDOS) *g* of the O p and the nearest‐neighbour surface Pt d states shown as a function of both energy (*E*−*E*_F_) and strain *σ* for the (2×2)‐OH/Pt(111) system. Each graph shows the spin‐up and spin‐down components in the *g*>0 and *g*<0 portions of that graph, respectively. Subsequent curves are offset vertically for clarity and were obtained using values of *σ*=0.95, 0.97, 0.99, 1.01, 1.03 and 1.05. The curves corresponding to *σ*=0.95 (1.05) curves lie closest to (farthest from) the line *g=*0. The oxygen atoms were bound a) across the reconstructed Pt(111) surface and b) in the on‐top position defined in Figure 1 of the unreconstructed surface.

Figure 10 shows the projected density of states (PDOS) *g* for the reconstructed and non‐reconstructed on‐top bound OH/Pt(111) systems. The valence orbitals of the OH groups can be seen in the compressed *σ*=−0.05 O PDOS curves of Figure 10 a in the interval $\left(E-E_{F}\right)\in \left[-8.0\right]$ eV. A significant delocalisation exists within these orbitals evidence the wide feature spanning$\left(E-E_{F}\right)\in \left[-5.0\right]$ eV. This delocalisation is removed for *σ*>−0.03 and replaced by a more localised orbital at *E−E*_F_ ≈−4.5 eV. Inspection of the non‐reconstructed PDOS curves in Figure 10 b indicates that the on‐top O binding to the reconstructed surface results in a predominantly more delocalised orbital than on‐top binding to the non‐reconstructed surface. The O−Pt bond lengths for on‐top binding to the reconstructed surface were 2.07–2.05 Å for $\sigma \in \left[-0.05,-0.03\right]$ . These are 0.05–0.03 Å longer than binding in the on‐top position at the same *σ* on the non‐reconstructed surface and are, in part, a consequence of the lower degeneracy of the delocalised orbitals seen on the reconstructed surface when compared to the more localised orbitals seen on the non‐reconstructed surface. Bond lengthening will also be a consequence of the increase in electron density accompanying hydrogenation. This is also evidence by comparing the O−Pt bond lengths of the OH/Pt(111) and those for on‐top bound O/Pt(111). In the latter case, the O−Pt bond length was 1.86 Å on the compressed (*σ*=−0.05) surface, decreasing to 1.85 Å when *σ*=+0.05. These comparisons show that the O−Pt bond length is largely governed by the binding position, degree of hydrogenation of the O and the reconstruction of the surface, and are only nominally determined by strain *σ*.

**Figure 11:**
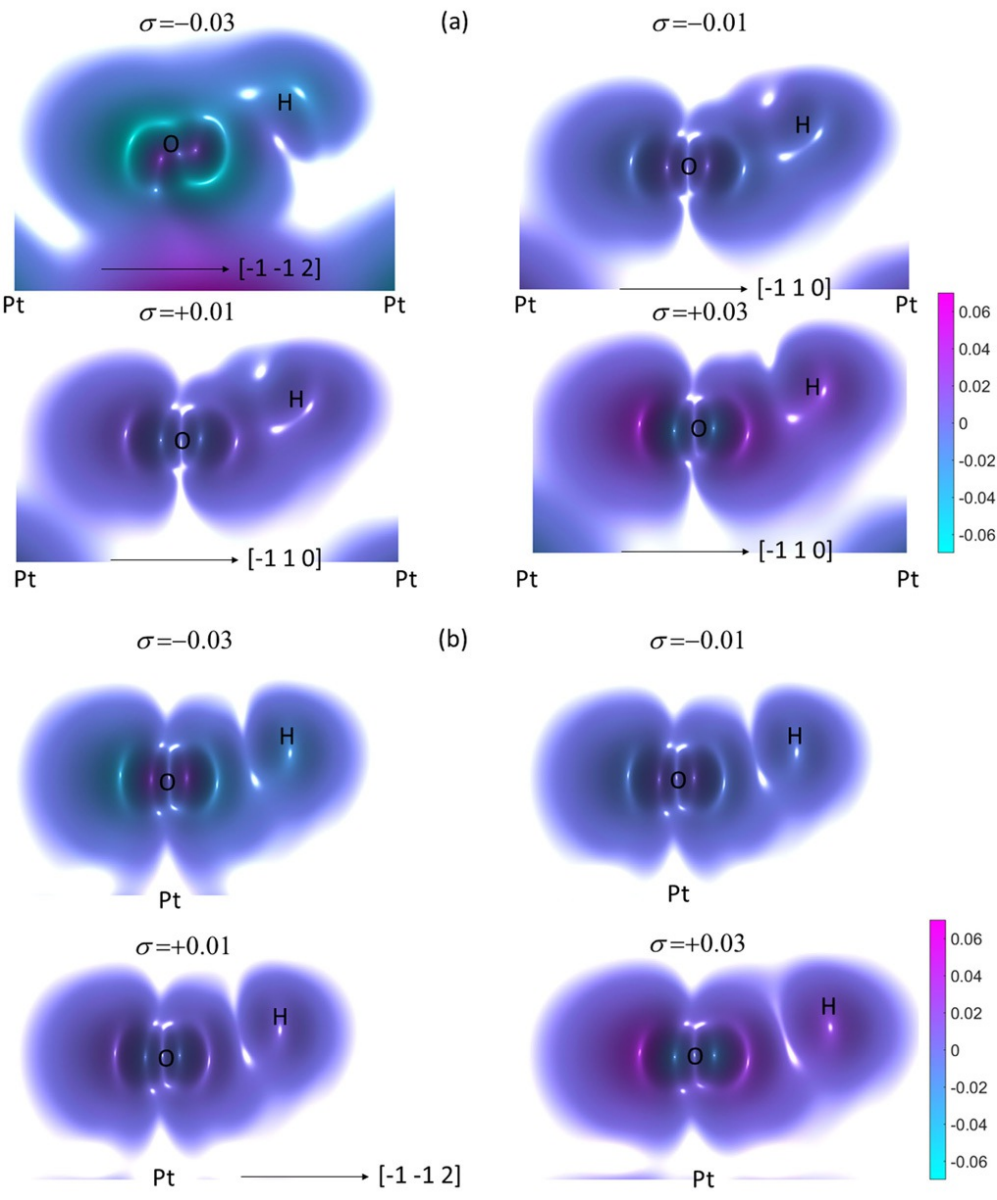
The difference electron density *ρ*_diff_ for the (2×2)‐OH/Pt(111) system for strain *σ*=−0.03, −0.01, +0.01 and +0.03. The oxygen atoms are bound a) across the reconstructed Pt(111) surface and b) at the on‐top sites of the unreconstructed surface. The legend shows the difference electron density in units of electrons/Ry^3^.

Figure 11 shows the difference electron density *ρ*_diff_ for the (2×2)‐OH/Pt(111) system with the oxygen atoms are bound across the a) reconstructed Pt(111) surface, and b) the on‐top sites of the unreconstructed surface. The character of the delocalised OH orbitals identified and discussed in the earlier discussion of the PDOS curves shown in Figure 10 are immediately apparent by comparing the *σ*=−0.03 panel of Figure 11 a with the more tensile (*σ*=−0.01,+0.01 and +0.03) panels in the same figure. In these latter panels, *ρ*_diff_ shows a localised accumulation around the O and H atoms, which increases with *σ*. This compares to the *σ*=−0.03 panel of Figure 11 a, where charge depletion is evident around the O and H atoms, and charge accumulation between the O atoms and the Pt surface layer. The increase in charge around the extended surface is consistent with the appearance of delocalised features in Figure 10. This mechanism is unique to binding in the on‐top position of the reconstructed surface compared to on‐top binding on the non‐reconstructed surface. This is evident by inspection of the *σ*=−0.03 panel of Figure 11 b, which does not show charge accumulation between the O atom and the surface Pt layer, and which shows a lower amount of charge depletion around the O and H atoms. The low symmetry, or ‘cusping’, of the feature around the O atom and between the O and H atoms in Figure 11 a is attributed to the differences in the angle of elevation of the OH bond (H atom) with respect to the surface Pt(111) plane between bridge‐bound OH (*σ*=0) and the on‐top bound OH (*σ*=−0.03). Removing this low symmetry from the O–H features introduces low symmetry features elsewhere in the panel showing *ρ*_diff_. Qualitatively, the characteristics of the feature—that it identifies a region of significant charge depletion—remains unchanged between the low and high symmetry presentations.

**Figure 12:**
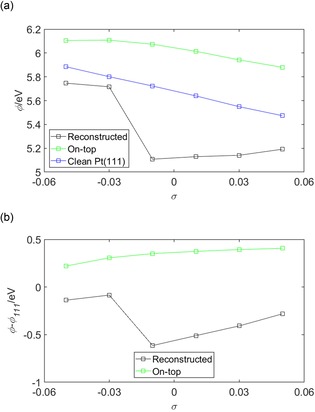
a) The work‐function *ϕ* of the (2×2)‐OH/Pt(111) system and b) the work function difference *ϕ*−*ϕ*_111_, where *ϕ*_111_ is the work of the clean Pt(111) surface, shown as a function of *σ*. The legends in (a) and (b) show the binding position of the oxygen atom either across the reconstructed Pt(111) surface or in the on‐top position defined in Figure 1 of the unreconstructed surface. Panel (a) also shows the curve corresponding to the clean Pt(111) surface.

Charge transfer will also contribute to changes in the work function *ϕ* of the surface. Figure 12 shows the work function *ϕ* and the work function difference *ϕ*−*ϕ*_111_, where *ϕ*_111_ is the work of the clean Pt(111) surface, of the reconstructed and on‐top bound (2×2)‐OH/Pt(111) systems. Changes in *ϕ* under strain have two components, one owing to Smoluchowski smoothing^54^ and the second due to the charge redistribution shown in terms of *ρ*_diff_ in Figure 11. In Figure 12 a, the *ϕ* for each of the reconstructed and on‐top bound OH/Pt(111) and the clean Pt(111) systems shows a general reduction as *σ* becomes increasingly tensile. This reduction is attributed to Smoluchowski smoothing^54^ and it's mechanism was discussed earlier when considering the work function behaviour of the O/Pt(111) system in Figure 6. A sharp transition is seen for the *ϕ* and *ϕ*−*ϕ*_111_ curves for the reconstructed O/Pt(111) system and shown in Figures 12 a and 12 b, respectively. This transition is attributed to the change in the binding position of the OH group and in the development of the Pt surface reconstruction. Changes in valency between the OH group and the Pt surface as the OH group moves between the on‐top and the bridge site may be anticipated significantly by the surface dipole layer and consequently the work function of the sample. These changes in the surface dipole layer were shown in terms of *ρ*_diff_ in Figure 11 a, particularly as the charge accumulation that develops between the O atoms and the Pt surface layer in the *σ*=−0.03 panel. This charge accumulation was discussed earlier; however, the work function behaviour presented in Figure 12 quantifies the effect of this accumulation.

## 3 Conclusions

In the current work, the effects of strain *σ* on the clean Pt(111) and the 0.25 ML O/Pt(111) and OH/Pt(111) surfaces have been investigated using density functional theory (DFT). The OH/Pt(111) surface has been seen to reconstruct under compressive strain (*σ*≤−0.03), and this reconstruction is accompanied by a change in the binding position of the OH between the on‐top and bridge sites. A similar phenomenon is seen on the O/Pt(111), where the O binding position changes from the bridge site (*σ*≤−0.03) to the FCC site (*σ*>−0.03).

The magnetisation of the clean Pt(111) surface and both the O/Pt(111) and OH/Pt(111) surfaces have been seen to be carried predominantly by the Pt d and O p states, and has been seen to increase as the strain *σ* becomes more tensile. The changes in magnetism have been accompanied by changes in the occupation of the magnetic quantum number resolved state populations *N*_*l,m*_ for both states, but without change to the angular quantum number resolved state populations *N_l_*. This is the same behaviour of the magnetisation for the ordered phases of Ni_*x*_Pt_1−*x*_ (*x=*0.25, 0.5, and 0.75)^8^ and both Pt_*x*_Fe_1−*x*_ and Pt_*x*_Co_1−*x*_.^9^ The charge transfers that accompany the changes in the magnetisation in both the current work and the previous studies of the bulk Ni_*x*_Pt_1−*x*_, Pt_*x*_Fe_1−*x*_ and Pt_*x*_Co_1−*x*_ systems show that the electronic changes are confined to intra‐orbital transitions for range of strains. This range was defined by considering the amount of lattice mismatch between the bulk alloy and a pure metal overlayer, which would be typical for a core–shell nanoparticle.

In the current work, phase transitions were seen for both the O/Pt(111) and OH/Pt(111) systems at the same strain *σ*=−0.03. This is suggestive that changes to the O−Pt bond are relatively insensitive to the presence of an H atom. Comparisons of the projected densities of states (PDOS) and the difference electron densities *ρ*_diff_ of both the O/Pt(111) and OH/Pt(111) systems show that, as expected, the O−Pt and OH−Pt interactions are substantially different. However, the observed alignment of the changes to these interactions under *σ* may prove useful in further studies, particularly if the O atom is patterned by a more complicated ligand than an H atom.

## Conflict of interest

*The authors declare no conflict of interest*.

## References

[1] Y. Nie , L. Li , Z. Wie , Chem. Soc. Rev. 2015, 44, 2168–2201. 25652755 10.1039/c4cs00484a

[2] J. Wu , H. Yang , Acc. Chem. Res. 2013, 46, 1848–1857. 23808919 10.1021/ar300359w

[3] V. R. Stamenkovic , B. Fowler , B. S. Mun , G. Wang , P. N. Ross , C. A. Lucas , N. M. Markovic , Science 2007, 315, 493–497. 17218494 10.1126/science.1135941

[4] V. R. Stamenkovic , B. S. Mun , M. Arenz , K. J. J. Mayrhofer , C. A. Lucas , G. Wang , P. N. Ross , N. M. Markovic , Nat. Mater. 2007, 6, 241–247. 17310139 10.1038/nmat1840

[5] L.-Å. Näslund , J. Chem. Phys. 2014, 140, 104701. 24628190 10.1063/1.4867535

[6] N. Agmon , Chem. Phys. Lett. 1995, 244, 456–462.

[7] C. Sachs , M. Hildebrand , S. Völkening , J. Wintterlin , G. Ertl , J. Chem. Phys. 2002, 116, 5759–5773.

[8] I. G. Shuttleworth , Chem. Phys. Lett. 2017, 689, 41–47.

[9] I. G. Shuttleworth , J. Phys. Chem. Solids 2018, 114, 153–162.

[10] I. G. Shuttleworth , Magnetochemistry 2016, 2, 39–1/12.

[11] M. Polak , L. Rubinovich , Surf. Sci. Rep. 2000, 38, 127–194.

[12] Y. Ma , P. B. Balbuena , Surf. Sci. 2008, 602, 107–113.

[13] Y. Ma , P. B. Balbuena , Surf. Sci. 2009, 603, 349–353.

[14] E. Fako , A. S. Dobrota , I. A. Pašti , N. López , S. V. Mentus , N. V. Skorodumovade , Phys. Chem. Chem. Phys. 2018, 20, 1524–1530. 29260157 10.1039/c7cp07276g

[15] L. Zhang , G. Henkelman , ACS Catal. 2015, 5, 655–660.

[16] M. Asano , R. Kawamura , R. Sasakawa , N. Todoroki , T. Wadayama , ACS Catal. 2016, 6, 5285–5289.

[17] I. G. Shuttleworth , Appl. Surf. Sci. 2016, 378, 286–292.

[18] I. G. Shuttleworth , Surf. Sci. 2017, 661, 49–59.

[19] T. M. Gür , Prog. Energy Combust. Sci. 2016, 54, 1.

[20] N. A. A. Rusman , M. Dahari , Int. J. Hydrogen Energy 2016, 41, 12108–12126.

[21] X. Yu , Z. Tang , D. Sun , L. Ouyang , M. Zhu , Prog. Mater. Sci. 2017, 88, 1–48.

[22] E. Zurek , Comments Inorg. Chem. 2017, 37, 78–98.

[23] G. Sudhapriyanga , A. T. Asvinimeenaatci , R. Rajeswarapalanichamy , K. Iyakutti , Acta Phys. Pol. A 2014, 125, 29–35.

[24] W. H. Zachariasen , C. E. Holley , J. F. Stamper , Acta Crystallogr. 1963, 16, 352.

[25] M. Bortz , B. Bertheville , G. Böttger , K. Yvon , J. Alloys Compd. 1999, 287, L4–L6.

[26] P. Vajeeston , P. Ravindran , B. C. Hauback , H. Fjellvåg , A. Kjekshus , S. Furuseth , M. Hanfland , Phys. Rev. B 2006, 73, 224102.

[27] T. Moriwaki , Y. Akahama , H. Kawamura , S. Nakano , K. Takemura , J. Phys. Soc. Jpn. 2006, 75, 074603.

[28] H. G. Schimmel , J. Huot , L. C. Chapon , F. D. Tichelaar , F. M. Mulder , J. Am. Chem. Soc. 2005, 127, 14348–14354. 16218629 10.1021/ja051508a

[29] J. L. Gland , B. A. Sexton , G. B. Fisher , Surf. Sci. 1980, 95, 587–602.

[30] M. Peuckert , H. P. Bonzel , Surf. Sci. 1984, 145, 239–259.

[31] H. Steininger , S. Lehwald , H. Ibach , Surf. Sci. 1982, 123, 1–17.

[32] G. N. Derry , P. N. Ross , Surf. Sci. 1984, 140, 165–180.

[33] G. N. Derry , P. N. Ross , J. Chem. Phys. 1985, 82, 2772–2778.

[34] D. H. Parker , M. E. Bartram , B. E. Koel , Surf. Sci. 1989, 217, 489–510.

[35] C. R. Parkinson , M. Walker , C. F. McConville , Surf. Sci. 2003, 545, 19–33.

[36] M. A. van Spronsen , J. W. M. Frenken , I. M. N. Groot , Nat. Commun. 2017, 8, 429. 28874734 10.1038/s41467-017-00643-z PMC5585323

[37] N. Materer , U. Starke , A. Barbieri , R. Döll , K. Heinz , M. A. Van Hove , G. A. Somorjai , Surf. Sci. 1995, 325, 207–222.

[38] P. Légaré , Surf. Sci. 2005, 580, 137–144.

[39] D. C. Ford , Y. Xu , M. Mavrikakis , Surf. Sci. 2005, 587, 159–174.

[40] Q. Pang , Y. Zhang , J.-M. Zhang , K.-W. Xu , Appl. Surf. Sci. 2011, 257, 3047–3054.

[41] M. T. M. Koper , R. A. van Santen , J. Electroanal. Chem. 1999, 472, 126–136.

[42] P. E. M. Siegbahn , U. Wahlgren , Int. J. Quant. Chem. 1992, 42, 1149–1169.

[43] F. McBride , A. Hodgson , Int. Rev. Phys. Chem. 2017, 36, 1–38.

[44] G. B. Fisher , B. A. Sexton , Phys. Rev. Lett. 1980, 44, 683–686.

[45] J. R. Creighton , J. M. White , Surf. Sci. 1982, 122, L648–L652.

[46] A. Michaelides , P. Hu , J. Chem. Phys. 2001, 114, 513–519.

[47] K. Bedürftig , S. Völkening , Y. Wang , J. Wintterlin , K. Jacobi , G. Ertl , J. Chem. Phys. 1999, 111, 11147–11154.

[48] M. T. M. Koper , T. E. Shubina , R. A. van Santen , J. Phys. Chem. B 2002, 106, 686–692.

[49] T. E. Shubina , M. T. M. Koper , Electrochim. Acta 2002, 47, 3621–3628.

[50] A. Panchenko , M. T. M. Koper , T. E. Shubina , S. J. Mitchell , E. Roduner , J. Electrochem. Soc. 2004, 151, A2016–A2027.

[51] P. Giannozzi , S. Baroni , N. Bonini , M. Calandra , R. Car , C. Cavazzoni , D. Ceresoli , G. L. Chiarotti , M. Cococcioni , I. Dabo , A. Dal Corso , S. Fabris , G. Fratesi , S. de Gironcoli , R. Gebauer , U. Gerstmann , C. Gougoussis , A. Kokalj , M. Lazzeri , L. Martin-Samos , N. Marzari , F. Mauri , R. Mazzarello , S. Paolini , A. Pasquarello , L. Paulatto , C. Sbraccia , S. Scandolo , G. Sclauzero , A. P. Seitsonen , A. Smogunov , P. Umari , R. M. Wentzcovitch , J. Phys. Condens. Matter 2009, 21, 395502–1/19. 21832390 10.1088/0953-8984/21/39/395502

[52] M. Methfessel , A. T. Paxton , Phys. Rev. B 1989, 40, 3616–3621. 10.1103/physrevb.40.3616 9992329

[53] The Pt.pbe-mt fhi.UPF, O.pbe-mt fhi.UPF and H.pbe-mt fhi.UPF pseudopotentials were used from the Quantum ESPRESSO pseudopotential data base: http://www.quantum-espresso.org/pseudopotentials.

[54] R. Smoluchowski , Phys. Rev. 1941, 60, 661–674.

